# Silent Manifestations of Disseminated Nocardia in a Renal Transplant Patient

**DOI:** 10.7759/cureus.65523

**Published:** 2024-07-27

**Authors:** Anam Ansari, Karim Dirani, Abubakar Malik, Sajith Matthews

**Affiliations:** 1 Internal Medicine, Wayne State University, Detroit, USA; 2 Internal Medicine, Wayne State University School of Medicine, Detroit, USA; 3 Internal Medicine, Detroit Medical Center, Wayne State University, Detroit, USA

**Keywords:** immunocompromised, musculoskeletal nocardiosis, renal transplant patient, pulmonary nocardia, disseminated nocardia

## Abstract

This case report describes a unique presentation of disseminated nocardiosis in a 45-year-old male transplant recipient who initially presented with acute left hip pain. Despite being asymptomatic for respiratory symptoms, the patient developed a fever and subsequently exhibited hypoxia. A diagnostic workup revealed a cavitary mass in the right upper lobe and multiple pulmonary nodules, confirming silent pulmonary nocardiosis. Concurrently, an MRI identified myositis and a possible abscess in the left hip musculature. Treatment involved a regimen including imipenem-cilastatin and linezolid, tailored for Nocardia species farcinica. This case underscores the importance of vigilant evaluation for metastatic infections in immunocompromised patients presenting with atypical symptoms, highlighting the necessity of imaging studies such as CT of the thorax for early detection of silent pulmonary involvement.

## Introduction

There are more than a hundred Nocardia species and among them, there are nine species that are involved with human disease. It is often difficult to identify which species is present in an infection and can take a significant amount of time to do so. In a developed country, the populations who are at high risk of developing disease secondary to a Nocardia infection are populations who are immunocompromised with deficient cell-mediated immunity. Examples of these patient populations would be patients with human immunodeficiency virus/acquired immunodeficiency syndrome, lymphoma, history of organ transplant, long-term glucocorticoid use, and immunomodulating therapeutic agent use. Disseminated Nocardia classically presents with skin or respiratory clinical manifestations in immunocompromised individuals. The onset of pneumonia due to Nocardia is acute in this patient population and typically presents with dyspnea, fatigue, fever, cough, hemoptysis, and chest pain [1].

Approximately half the patients with pulmonary nocardiosis present with disseminated disease, most commonly the central nervous system and rarely the musculoskeletal system [1]. However, there is a paucity of literature documenting silent pulmonary nocardiosis with a coexisting presentation of musculoskeletal symptoms, as well as coexisting pulmonary and musculoskeletal nocardiosis. We present a rare case of a transplant patient presenting with silent pulmonary and symptomatic musculoskeletal nocardiosis. About half the patients with pulmonary involvement have disseminated disease, such as spread to the skin, supporting structures, muscles, bones, eyes, and most commonly the brain. CT lung findings typically show pulmonary nodules and cavitary lesions [2]. Disease in the integumentary system can present in different forms such as cellulitis, lymphocutaneous disease, and actinomycetoma, which typically do not result in disseminated infection [1].

## Case presentation

A 45-year-old male, with a history of deceased donor transplant three years prior, presented to the emergency department with acute onset left-sided hip pain of one-week duration and was admitted for possible bursitis. His pain radiated to his lower back and posterior left thigh. He was on immunosuppressive therapy with prednisone, tacrolimus, and mycophenolate. Physical examination (only hip and pulmonary exam) showed bilateral lower extremity muscle strength five out of five, and limited range of motion of hip movement with pain elicited with palpation. Respiratory and integumentary physical examinations were within normal limits. Complete blood count with differential showed leukocytosis with a neutrophilic predominance. CT of the abdomen and pelvis obtained in the emergency department showed mild left hip osteoarthrosis. We admitted him for acute left hip pain due to possible bursitis and lumbar radiculopathy.

**Figure 1:**
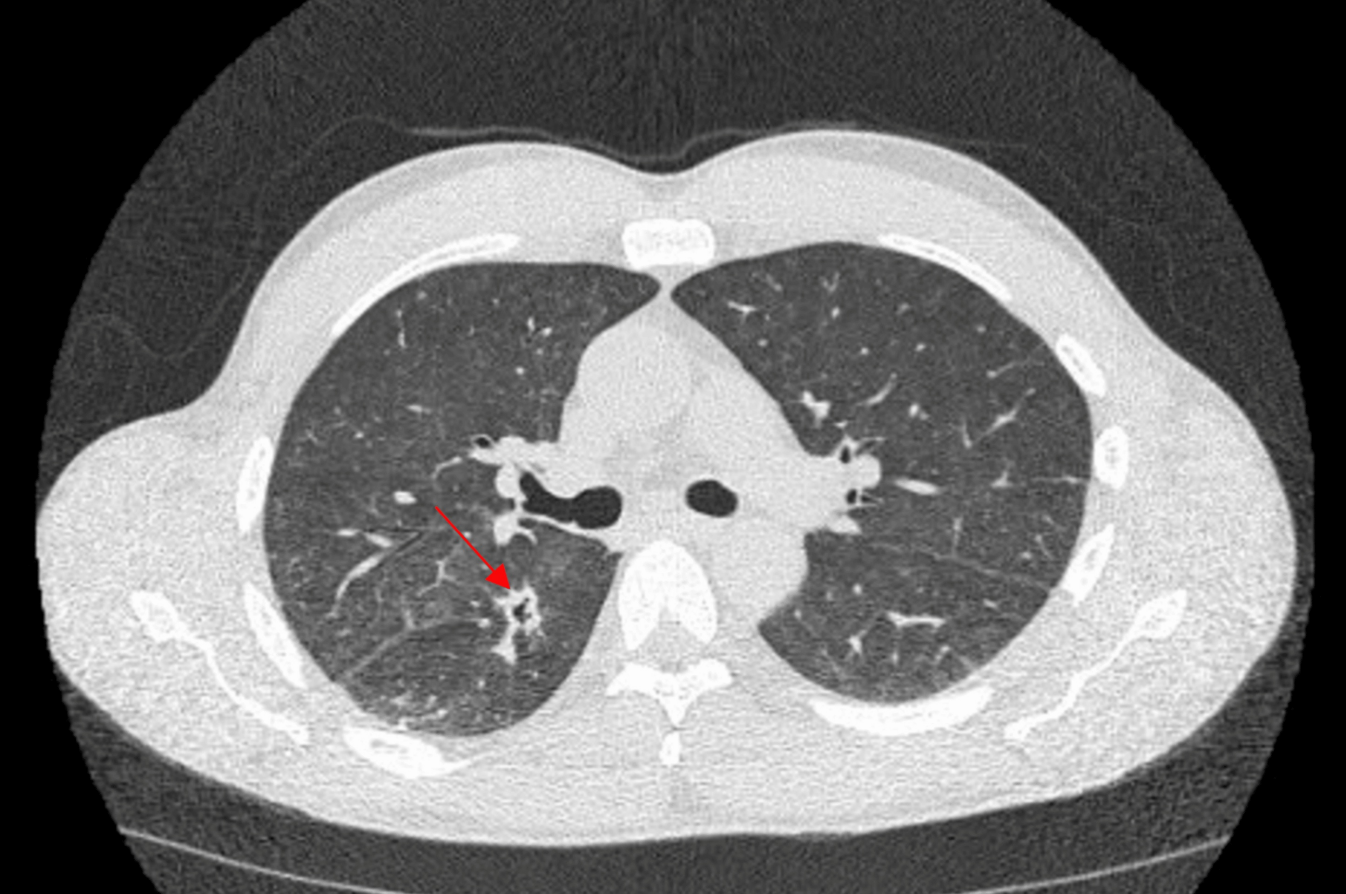
CT of the chest without contrast (axial image) at the level of the right mainstem bronchus demonstrates a cavitary lesion in the right lower lobe.

**Figure 2:**
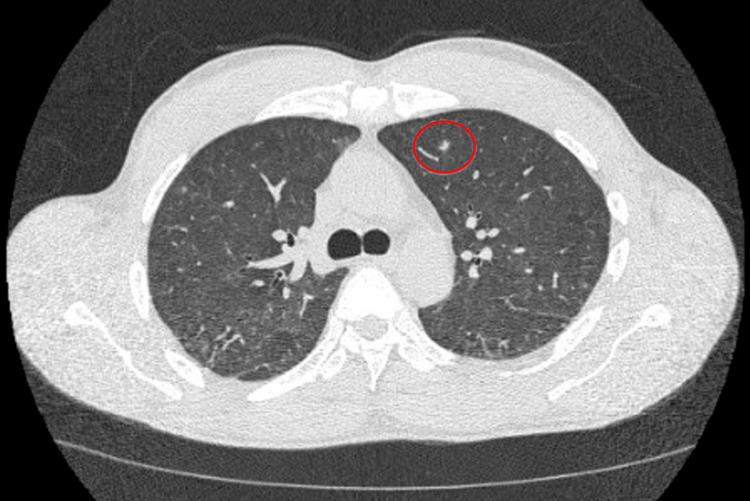
CT of the chest without contrast (axial slice) at the level of the carina demonstrates a solitary left upper lobe solid pulmonary nodule (6 mm).

On day two of admission, our patient developed a fever of 39.4°C. His only complaint was hip pain, and now he developed a fever, making us suspicious that he may have an infection in his left hip joint such as an abscess or osteomyelitis. We ordered a septic workup, which included blood cultures, and he was started on vancomycin and cefepime. MRI of the left hip was also ordered to evaluate for infection. Furthermore, his chest X-ray was normal. On day four of admission, the blood culture grew branching, partially acid-fast gram-negative bacilli. Vancomycin and cefepime were discontinued and the patient was started on meropenem 1 gram every eight hours to treat a likely Nocardia infection. On day five of admission, the patient’s oxygen saturation decreased to the 80s, and he was started on supplemental oxygen via nasal cannula. Despite the patient denying any symptoms of cough or shortness of breath, his oxygen saturation would intermittently decline. A CT of the thorax without contrast (Figures 1, 2) was ordered and it showed interval development of cavitary mass in the inferior aspect of the right upper lobe and innumerable bilateral new pulmonary nodules.

**Figure 3:**
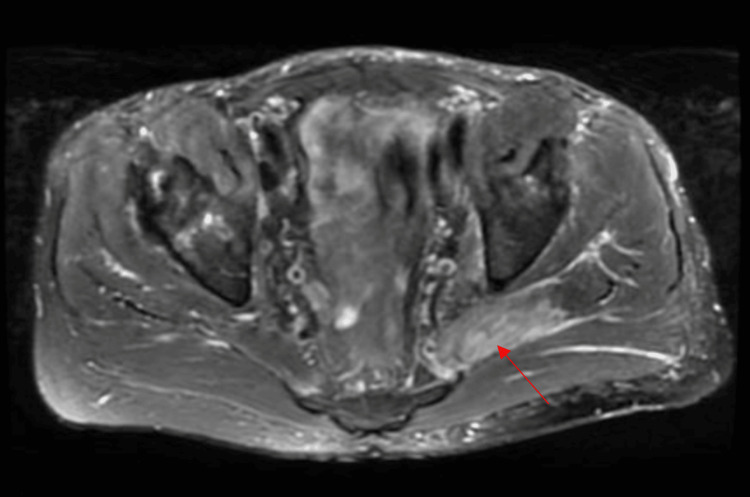
MRI of the pelvis/hip, T2 fat saturation axial image, at the level of the lower pelvis demonstrates a T2 signal in the left piriformis compatible with intramuscular edema measuring 1.5 x 0.4 cm.

On day six of admission, the patient was diagnosed with disseminated nocardiosis. Meropenem was discontinued, and the patient was started on imipenem-cilastatin 1 gram every eight hours and linezolid 600 milligrams twice daily for Nocardia coverage. On day eight of admission, an MRI of the pelvis/hip (Figure 3) showed the following findings: myositis involving the left piriformis, gluteus medius, and minimus muscles. Possible intramuscular abscess of the left piriformis. This indicates that there is dissemination to bone as well. MRI of the brain was done to evaluate for metastatic infection and it was negative. Due to a severe infection, he was discharged on ceftriaxone 2 grams daily and linezolid 600 milligrams twice daily with a plan for a total six-month course of antibiotics. Upon outpatient follow-up with infectious disease, the patient had marked improvement in his symptoms and further noted that his hip pain was infrequent now. The blood culture resulted in Nocardia species farcinica.

The patient followed up in the infectious disease (ID) clinic every one to two months after discharge. Seven months after his hospitalization was his last clinic visit with ID. During that visit, it was noted that he had no symptoms and had completed his antibiotic course.

## Discussion

A retrospective study of 36 patients diagnosed with pulmonary nocardiosis showed that 91.7% of patients presented with cough, 77.8% with fever, 72.2% with expectoration, 66.7% with breathlessness, and 50% with malaise [3]. The report noted that cough, fever, and sputum production were the most common symptoms [3]. Our patient had a rare case of asymptomatic pulmonary nocardiosis where the symptoms were predominantly musculoskeletal in an immunocompromised patient. There were no presenting respiratory symptoms, no physical exam findings, and no chest X-ray findings that were indicative of pulmonary nocardiosis. Based on our literature review, there have been documented cases of asymptomatic pulmonary nocardiosis in patients initially diagnosed with musculoskeletal infections such as septic arthritis or muscle abscess [4,5]. These occurrences are rare, as pulmonary symptoms typically manifest in cases of pulmonary nocardiosis, as seen in a case involving osteomyelitis and disseminated nocardiosis, where one of the symptoms the patient initially presented with included cough [6] and another case where the patient had progressive bilateral lower extremity pain and swelling along with shortness of breath [7].

The diagnosis of pulmonary nocardiosis is often delayed and the mortality rate of disseminated nocardiosis is high [8]. This highlights the importance of assessing for metastatic pulmonary infection in a patient who has nocardiosis or even if there is suspicion of nocardiosis, especially in an immunocompromised patient. This case was rare, as his only complaint on admission was musculoskeletal, and he did not endorse any respiratory symptoms even when he was incidentally found to be hypoxic during routine vital checks on his fifth day of hospitalization. This case emphasizes that if we suspect a patient has Nocardia bacteremia, we do not need to wait for the patient to develop respiratory symptoms or physical exam findings such as hypoxia to investigate for silent pulmonary nocardiosis with CT imaging. It is also to be noted that while the patient had a silent pulmonary infection, there was no metastatic infection to the brain, and pursuing an MRI of a brain in such a setting for every case remains unknown.

## Conclusions

This case illustrates the importance of getting a complete radiological workup of the area of pain, along with CT of the thorax and MRI of the brain in a timely manner when we have any suspicion for an immunocompromised patient to have disseminated nocardiosis. Radiological imaging is important in helping us determine the involved organs, and the degree of dissemination will guide us on how many antibiotics to use along with their duration.
